# Evaluation of Interpolation Effects on Upsampling and Accuracy of Cost Functions-Based Optimized Automatic Image Registration

**DOI:** 10.1155/2013/395915

**Published:** 2013-08-01

**Authors:** Amir Pasha Mahmoudzadeh, Nasser H. Kashou

**Affiliations:** Biomedical Imaging Laboratory, Wright State University, Dayton, OH 45435, USA

## Abstract

Interpolation has become a default operation in image processing and medical imaging and is one of the important factors in the success of an intensity-based registration method. Interpolation is needed if the fractional unit of motion is not matched and located on the high resolution (HR) grid. The purpose of this work is to present a systematic evaluation of eight standard interpolation techniques (trilinear, nearest neighbor, cubic Lagrangian, quintic Lagrangian, hepatic Lagrangian, windowed Sinc, B-spline 3rd order, and B-spline 4th order) and to compare the effect of cost functions (least squares (LS), normalized mutual information (NMI), normalized cross correlation (NCC), and correlation ratio (CR)) for optimized automatic image registration (OAIR) on 3D spoiled gradient recalled (SPGR) magnetic resonance images (MRI) of the brain acquired using a 3T GE MR scanner. Subsampling was performed in the axial, sagittal, and coronal directions to emulate three low resolution datasets. Afterwards, the low resolution datasets were upsampled using different interpolation methods, and they were then compared to the high resolution data. The mean squared error, peak signal to noise, joint entropy, and cost functions were computed for quantitative assessment of the method. Magnetic resonance image scans and joint histogram were used for qualitative assessment of the method.

## 1. Introduction 

### 1.1. Interpolation

**Figure 1:**
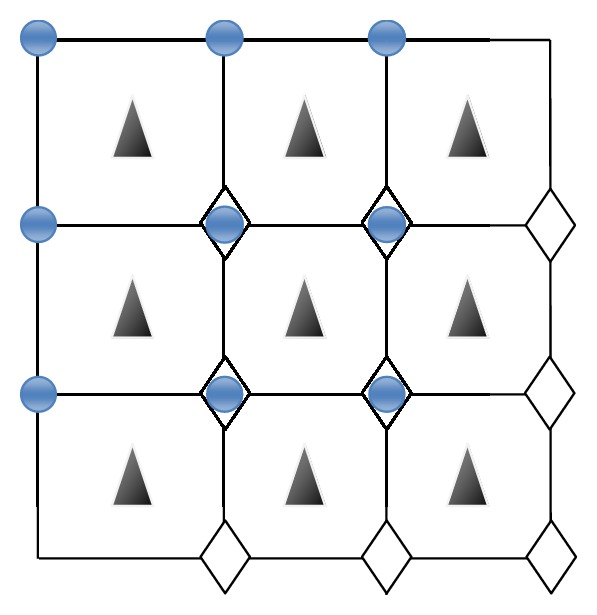
Scheme for interpolation. Straight line shows the original HR grid, circle shows the reference HR pixels, and a diamond and a triangle are shifted version of HR pixels.

 One of the most important parts of designing a registration algorithm is choosing a good interpolation function in order to increase the accuracy of registration. Also, Interpolation is required if the fractional unit of the motion is not matched and located on high resolution (HR) grid. One of the ways by which we can help physicians in coming up with a better diagnosis and treatment is improving the resolution of images. One scheme for the interpolation step is shown in Figure 1. Here, a circle shows the reference HR image, and a diamond and a triangle represent a shifted HR pixel. For instance, if the image is downsampled by a factor of 4, a diamond has (0.25, 0.25) subpixel shift for the vertical and horizontal directions and a triangle has a shift that is less than (0.25, 0.25). In Figure 1, a triangle is not placed on the HR grid and it needs interpolation, but a diamond does not need interpolation.

Therefore, some interpolation approaches are proposed to overcome the problem of low resolution in medical imaging. Magnetic resonance imaging (MRI) is an invaluable modality in the medical field. Particularly, neuroimaging with MRI helps physicians to study the internal structure and functionality of the human brain. In these cases, high resolution and isotropic images are important because higher isotropic resolution could theoretically reduce partial volume artifacts, leading to better accuracy/precision in deriving volumetric measurement and decreasing considerable errors in the registration [1]. Clinically, acquiring a fully isotropic 3D image set is not feasible because of time, motion artifacts, and PSNR factors. Thus, typically, in 3D MR data, the in-plane direction has higher resolution than the slice direction (*Z*-axis). In this case, invaluable information will be lost in the latter direction. Our objective is to recover and fill in this missing information in order to enable the physicians to have a more accurate perspective of the underlying structure available in the data by optimizing the choice of interpolation techniques.

The study of interpolation approaches dates back to the 1980s [2]. In which a great diversity of techniques can be found in the literature. For example, B-splines were sometimes referred to as cubic splines [3], whereas cubic interpolation was also known as cubic convolution [4–6] and as high resolution spline interpolation [2]. Eight interpolation algorithms are reviewed in the following sections. We first present cubic Lagrangian, quintic Lagrangian, and heptic Lagrangian. Then, we explain a nearest neighbor interpolation approach which is associated with strong aliasing and blurring effect. Next, discussions of the trilinear interpolation approach as well as B-spline 3rd order, B-spline 4th order, and windowed Sinc are explained. Finally, we discuss and evaluate the performance of these interpolation algorithms in order to find the best interpolation method for upsampling of 3D MR images. Different 2D interpolation approaches exist in medical imaging [4]. However, in this paper, we compare the performance (quality and quantity) of eight common interpolation approaches on 3D data.

#### 1.1.1. Lagrange Interpolation

 Lagrange interpolation is a famous, classical technique for interpolation. The Lagrange interpolation is a way to pass a kernel of degree *N* − 1 through *N* × *N* points and is defined in *X*-direction (for 2D image, adds *Y*-direction, and for 3D image adds *Y*- and *Z*-directions) [5, 6, 8–13],

(1) $$\begin{array}{c} {\text{Lagra}}_{hn(x)}=\left\{\begin{array}{cc} \prod_{j=0,j-\left(N/2\right)+1\neq n}^{N-1}\frac{n-i-x}{n-i}, & n-1\leq x<n\\ 0, & \text{elsewhere}, \end{array}\right\}, \end{array}$$

where *i* = *j* − (*N*/2) + 1 and *n* ∈ {−(*N*/2) + 1, −(*N*/2) + 2,…, *N*/2}  are the Lagrange kernels. The Lagrange kernel for *N* = 1 equals the nearest neighbor interpolation. In this case, *N* = 2 equals the linear interpolation. The Lagrange kernels for *N* = 4 and *N* = 5 supporting points result in cubic and quartic polynomials, respectively, and are shown as follows

(2) $$\begin{array}{c} {\text{Lagra}}_{h4(x)}=\{\begin{array}{cc} \frac{1}{2}x^{3}-x^{2}-\frac{1}{2}x+1, & \text{for}\;\;0\leq x<1,\\ \frac{-1}{6}x^{3}+x^{2}-\frac{11}{6}x+1, & \text{for}\;\;1\leq x<2,\\ 0, & \text{elsewhere}, \end{array}\\ {\text{Lagra}}_{h5(x)}\\ \;=\{\begin{array}{cc} \frac{1}{4}x^{4}-54x^{2}+1, & \text{for}\;\;0\leq x<\frac{1}{2},\\ \frac{-1}{6}x^{4}+\frac{5}{6}x^{3}-\frac{5}{6}x^{2}-\frac{5}{6}x+1, & \text{for}\;\;\frac{1}{2}\leq x<\frac{3}{2},\\ \frac{1}{24}x^{4}-\frac{5}{12}x^{3}+\frac{35}{24}x^{2}-\frac{25}{12}x+1, & \text{for}\;\;\frac{3}{2}\leq x<\frac{5}{2},\\ 0, & \text{elsewhere}. \end{array} \end{array}$$

#### 1.1.2. Nearest Neighbor Interpolation

 Nearest neighbor interpolation (also known as zero-order interpolation) is the simplest method, and strong aliasing and blurring effects are associated with this interpolation [14]. The local 1-point Lagrange interpolation is equivalent to the nearest neighbor interpolation, defined by

(3) $$\begin{array}{c} w\left(x,n\right)=\{\begin{array}{cc} 1, & \text{for}\;\;n-\frac{1}{2}\leq x<n+\frac{1}{2},\\ 0, & \text{otherwise}. \end{array} \end{array}$$

The images when scaled up in size may look very blocky. Likewise, the local 2-point Lagrange interpolation is equivalent to the linear interpolation, defined by

(4) $$\begin{array}{c} w\left(x,n\right)=\{\begin{array}{cc} 1-\left|x-n\right|, & \text{for}\;\;n-1\leq x<n+1,\\ 0, & \text{otherwise}. \end{array} \end{array}$$

#### 1.1.3. Trilinear Interpolation

**Figure 2:**
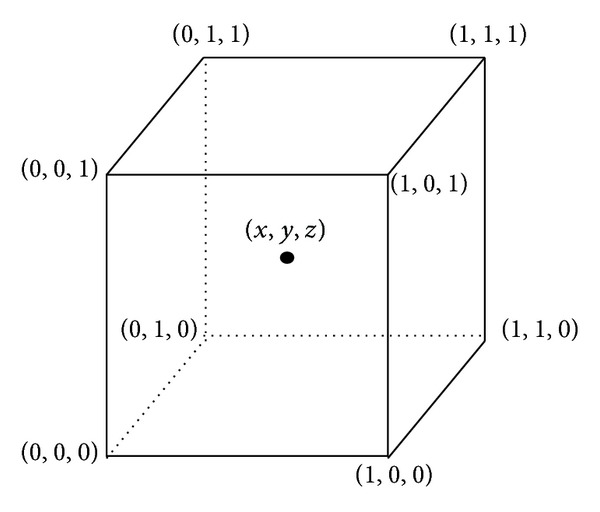
Trilinear interpolation computes values located between existing *voxel* values by linearly weighting the eight closest neighboring values (obtained from National Institutes of Health Center for Information Technology, Rockville, MD, USA).

Trilinear interpolation calculates values placed between existing *voxel* values by linearly weighting the eight closest neighboring values. In other words, trilinear is the name given to the process of linearly interpolating points within a 3D box, given the values at the vertices of the box (see Figure 2) [15].

The known values at each vertex are indicated as *V*000, *V*100, *V*010,…, *V*111, and the unknown value is calculated by merging the known corner values weighted by their distance from the point (*x*,*y*, *z*) within the cube.

#### 1.1.4. B-Spline Interpolation

 B-spline interpolation uses weighted *voxel* values in a wider neighborhood compared to trilinear interpolation, but both the B-spline and trilinear kernels are symmetrical and separable. The place of the neighboring points as control points relates to B-spline interpolation and combines the intensity values at these places using a set of polynomial basis according to (5) [16].

Equation (5) shows *k*-order B-spline with *n* + 1 control points (*P*1, *P*2,…, *Pn*),

(5) $$\begin{array}{c} P\left(t\right)=\sum_{i=1}^{n+1}N_{i,k}P_{i},\;t_{\min}\leq t<t_{\max}. \end{array}$$

In (5), *N*_*i*,*k*_ are the polynomial functions of order *k* (degree *k* − 1), and *n* is the number of control points; *k* must be at least 2 (linear) and less than *n* + 1.

*P*(*t*) is validly defined for *t*_min⁡_ ≤ *t* < *t*_max⁡_, where *t*_min⁡_ = *t*_*k*_ and *t*_max⁡_ = *t*_*n*+2_. A knot vector (*t*_1_, *t*_2_,…, *t*_*k*+(*n*+1)_) must be determined. This specifies the values of *t* at which the pieces of curve join, like knots joining bits of string. It is important to note that the degree of the weighting polynomial (the order of the curve) is not dependent on the number of control points, *n* [17].

The weighting polynomial can be recursively defined by the following equation [18]:

(6) $$\begin{array}{c} N_{i,1}\left(t\right)=\{\begin{array}{cc} 1, & t_{i}\leq t<t_{i+1},\\ 0, & \text{otherwise}, \end{array}\\ N_{i,k}=\frac{t-t_{i}}{t_{i+k-1}-t_{i}}N_{i,k-1}\left(t\right)+\frac{t_{i+k}-t}{t_{i+k}-t_{i+1}}N_{i+1,k-1}\left(t\right). \end{array}$$

**Figure 3:**
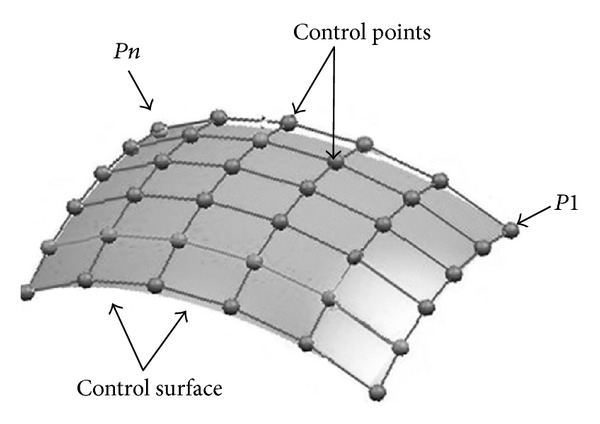
B-spline interpolation. There are *n* control points (*P*1, *P*2,…, *Pn*). The sequence of the control point is called a *control surface* (adapted from National Institutes of Health Center for Information Technology, Rockville, MD, USA).

In (6), *t*(*i*) represents an index that refers to the control points and *t*(*i*) are generally referred to as knot points (see Figure 3). The series of control point is defined as a control surface. This indexing scheme allows one to weight different control points more than other control points by using it once during the computation. Typically, the first and last control points are weighted more heavily than the internal points to give a smooth interpolating curve. Generally, the shape of the curve (*N*_*i*,*k*_) is specified by the relative spacing between the knots (*t*_0_, *t*_1_,…, *t*_*n*_). The sequence (*t*_0_, *t*_1_,…, *t*_*n*_) is called knot vector. Knot vectors are generally placed into one of three categories: uniform, nonuniform, and open uniform. Uniform knot vectors are the vectors for which *t*_*i*+1_ − *t*_*i*_ = const, for example, [0, 1, 2, 3, 4, 5, 6, 7, 8, 9]. Nonuniform knot vectors are a general case; the only constraint is the standard *t*_*i*_ ≤ *t*_*i*+1_, for example, [0.2, 05, 0.8, 0.8, 0.8, 1.1, 1.1, 2.2, 2.7, 3.4]. The optimized automatic image registration (OAIR) method uses the open uniform knot vectors for computing B-spline. Open uniform knot vectors are uniform knot vectors which have *k*-equal knot values at each end as [3, 5, 9, 16–19]

(7) $$\begin{array}{c} t_{i}=t_{0},\;i<k,\\ t_{i+1}-t_{i}=\text{const},\;\;k-1\leq i<n+1,\\ t_{i}=t_{k+n},\;\;i\geq n+1, \end{array}$$

for example, [0, 0, 0, 1, 2, 3, 4, 4, 4] (here *k* = 3, *n* = 5). 

#### 1.1.5. Windowed Sinc Interpolation

 This interpolation function has minimum aliasing artifacts in contrast to linear interpolation. Sinc function can be windowed more generally to yield [5, 10] the following

(8) $$\begin{array}{c} \text{sinc}\left(x\right)=\{\begin{array}{cc} \frac{\sin\left(x\right)}{x}, & \forall x\neq 0,\\ 1, & x=0. \end{array} \end{array}$$

Think of an image data set comprising a 3D matrix *voxel* with intensities *I*(*x*, *y*, *z*), specified by integer position coordinates (*x*, *y*, *z*). If one wants to calculate the intensity value at an interior point defined by noninteger coordinates (*x*, *y*, *z*), this can be obtained by the following equation [5]:

(9) I(x,y,z)=∑X∑Y∑ZI(X,Y,Z)sinc⁡(π(x−X))    ×sinc⁡(π(y−Y))sinc⁡(π(z−Z)).

For satisfying (9), two limiting conditions are required:

- *I*(*x*, *y*, *z*) must be band limited. Put differently, the function must have *Fourier transform F*{*I*(*x*, *y*, *z*)} = *I*(*f*) = 0 for |*f*| > *B* for some maximum frequency *B* > 0;
- the sampling rate—*f*_*s*_ must be greater than twice the bandwidth, for example, *f*_*s*_ > 2*B*.

The following section discusses the registration process and explains how interpolation is involved with registration.

### 1.2. Image Registration Algorithms

 Image registration methods in medical imaging seek to align two or more images and can be applied in the same modality on the same patient for the purpose of monitoring and quantifying disease progression over time. Registration can also be applied across different modalities, which is useful for correction of different patient positions across scans, for instance, aligning positron emission tomography (PET) data to an MRI image. Also, image registration can be used on the different patients, which is useful for studies of variability between subjects. Image registration is classified into the following categories and depends on several factors: image modalities (MRI, PET, CT, etc.), the subject of registration (a single person or different persons), the object of registration (head or heart), the image dimensionality (e.g., 2D, 3D, and 4D), and geometrical transformation (affine, rigid, projective, etc.). 

This study examines 3D affine registration of brain images using *voxel* intensities similarity measures such as normalized mutual information (NMI), normalized cross correlation (NCC), least squares (LS), and correlation ratio (CR). More explicitly, if a target image is resampled to match a reference image, the image intensities at each *voxel* should be similar in the two images. In fact, when utilizing an intensity-based cost function, it is essential to repeatedly resample one of the images to match the others at several various resolutions, while searching for the min cost function. This resampling process requires interpolation during the registration process [19]. In the optimized automatic image registration (OAIR) method, interpolation involves resampling of anisotropic *voxels* in the *z*-direction into isotropic cubic *voxels*. Also, it is important to note that in the OAIR method, the interpolation technique utilized for registration does not necessarily need to be the same interpolation technique used during registration to compute a final image using the optimal parameters.

In this paper, we are focusing on the effect of interpolation technique and cost function used for intensity-based registration. The following sections are organized as follows, we first give some background and provide a means of defining the critical components involved in image registration and establish a theoretical framework.

#### 1.2.1. Geometric Transformation

 When registering images, one should specify a geometric transformation that specially aligns one image to another. The common transformations can be classified as rigid, affine, and projective. Rigid transformation can be defined as a simple transformation that includes only translation and rotation. The projective transformation is the most general transformation and maps lines to lines (but does not necessarily preserve parallelism). An affine transformation includes scaling, rotation, translation, shearing, and reflection. There are several scanner-produced errors that can result in skewing or scaling terms, and affine transformations are applied to overcome these problems. 

**Figure 4:**
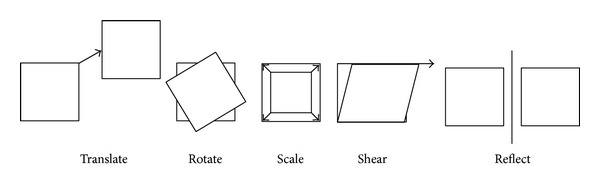
The five basic affine transformations are translate, rotate, scale, shear, and reflection. Translate moves a set of points a fixed distance in *x*- and *y*-directions, Rotate rotates a set of points about the origin, Scale scales a set of points up or down in *x*- and *y*-directions, and Shear offsets a set of points a distance proportional to their *x*- and *y*-coordinates. Reflection produces a mirror image of a set of points in *x*- or *y*-directions.

An affine transformation maps straight lines to straight lines and keeps the parallelism of lines, but not their lengths or their angles. Changing scaling and shearing factors for each image dimension will extend the degree of freedom (DOF, the number of independent pieces of information that go into the estimate of a parameter) of the rigid transformation [20–27].  Figure 4 shows the five basic components of affine transformations. The following matrices constitute the basic affine transforms in 3D, addressed in homogeneous form.

*Translation*. Translate a point in the *xyz*-plane to a new place by adding a vector (*t*_*x*_, *t*_*y*_, *t*_*z*_). **x**′ = *x* + *t*_*x*_, **y**′ = *y* + *t*_*y*_, and **z**′ = *z* + *t*_*z*_. **P**′ represents scaled matrices: **P**′ = **T** **P**, where

(10) $$\begin{array}{c} P^{\prime }=\left[\begin{array}{c} x^{\prime }\\ x^{\prime }\\ y^{\prime }\\ 1 \end{array}\right],\;\;P\;=\left[\begin{array}{c} x\\ y\\ z\\ 1 \end{array}\right], \end{array}$$

(11) $$\begin{array}{c} T\left(t_{x},t_{y},t_{z}\right)\text{:}\left[\begin{array}{cccc} 1 & 0 & 0 & t_{x}\\ 0 & 1 & 0 & t_{y}\\ 0 & 0 & 1 & t_{z}\\ 0 & 0 & 0 & 1 \end{array}\right]. \end{array}$$

  

*Scaling*. Scaling is making the new scale of a coordinate direction p times larger. Scaling is applied to all axes, each with a different scaling factor (*s*_*x*_, *s*_*y*_, *s*_*z*_). **x**′ = *s*_*x*_ × *x*, **y**′ = *s*_*y*_ × *y* and **z**′ = *s*_*z*_ × *z*. **P**′ represents of scaled matrices:

**P**′ = **S** **P**,

(12) $$\begin{array}{c} S\left(s_{x},s_{y},s_{z}\right)\text{:}\left[\begin{array}{cccc} s_{x} & 0 & 0 & 0\\ 0 & s_{y} & 0 & 0\\ 0 & 0 & s_{z} & 0\\ 0 & 0 & 0 & 1 \end{array}\right]. \end{array}$$

*Rotation*. If a point (*x*, *y*, *z*) is rotated an angle *θ* about the coordinate origin to become a new point (**x**′,**y**′, **z**′), the three basic rotations in 3D can be defined as follows:

Rotation  about  the *x*-axis:

(13) $$\begin{array}{c} \left[\begin{array}{c} x^{\prime }\\ y^{\prime }\\ z^{\prime }\\ 1 \end{array}\right]=\left[\begin{array}{cccc} 1 & 0 & 0 & 0\\ 0 & \cos\theta & -\sin\theta & 0\\ 0 & \sin\theta & \cos\theta & 0\\ 0 & 0 & 0 & 1 \end{array}\right]\left[\begin{array}{c} x\\ y\\ z\\ 1 \end{array}\right]. \end{array}$$

Rotation  about  the *y*-axis:

(14) $$\begin{array}{c} \left[\begin{array}{c} x^{\prime }\\ y^{\prime }\\ z^{\prime }\\ 1 \end{array}\right]=\left[\begin{array}{cccc} 1 & \cos\theta & \sin\theta & 0\\ 0 & 0 & 0 & 0\\ 0 & -\sin\theta & \cos\theta & 0\\ 0 & 0 & 0 & 1 \end{array}\right]\left[\begin{array}{c} x\\ y\\ z\\ 1 \end{array}\right]. \end{array}$$

Rotation  about  the *z*-axis:

(15) $$\begin{array}{c} \left[\begin{array}{c} x^{\prime }\\ y^{\prime }\\ z^{\prime }\\ 1 \end{array}\right]=\left[\begin{array}{cccc} \cos\theta & -\sin\theta & 0 & 0\\ \sin\theta & \cos\theta & 0 & 0\\ 0 & 0 & 1 & 0\\ 0 & 0 & 0 & 1 \end{array}\right]\left[\begin{array}{c} x\\ y\\ z\\ 1 \end{array}\right]. \end{array}$$

There are several reasons for using homogeneous coordinates, including the ability to apply all four transformations multiplicatively. In view of the fact that transformation combinations (shearing, scaling, and rotation) are all multiplicative transforms, only translation is an additive transform.

Next, the following cost functions are defined and described: LS, NCC, CR, and NMI. Furthermore, an overview of the literature on their use in registration for medical applications is included. Among these cost functions, the NMI-based registration has become commonplace in many medical applications [28]. 

#### 1.2.2. Cost Functions

 The cost function or similarity measure evaluates the similarity between two images. In this section, the behaviors of four commonly used cost functions will be examined.

*Least Squares (LS).* The least squares method measures the average of the squared difference in image intensities [29]:

(16) $$\begin{array}{c} f=\frac{\sum_{i=1}^{N}{\left\{R-I\right\}}^{2}}{N}, \end{array}$$

where *R* is the reference image, *I* is the input image, *N* is the number of values over which the sum is performed, and *f* is the least square. When two images differ only by Gaussian noise, the least squares will be the optimum cost function. Images of two different modalities such as MRI and PET will never differ by only Gaussian noise. Due to patient motion, even two images of the same modality, such as two MRI images, will rarely only differ by Gaussian noise. The effectiveness of LS will be extremely decreased by a small number of *voxels* having considerable intensity differences.

*Correlation Ratio (CR).* The main principle of the correlation ratio method is to calculate a “similarity measure” between a reference image and an input image and search for a spatial transformation *T* and an intensity mapping *f* such that by dis-replacing *R* and remapping its intensities, the resulting image *f*(*R* × *T*) can be seen as equivalent as possible to *I*. This can be obtained by minimizing the following CR function [30]:

(17) $$\begin{array}{c} \text{minimizing}\left(T,f\right)\;\;\text{of}\;\;\sum_{k}\left\{I\left(x_{k}\right)-f\left(R\left(T\left(x_{k}\right)\right)\right)\right\}, \end{array}$$

which integrates over the *voxel* positions in the image *I*. The minimum and maximum values for the CR are 0 and 1, respectively. The CR can be applied in multimodal image registration involving positron emission tomography (PET), MRI, and computed tomography (CT) images, providing a good tradeoff between accuracy and robustness [31]. 

*Normalized Cross-Correlation (NCC).* The cross-correlation function works very well for aligning images of the same modality. Cross-correlation function is defined by the following equation:

(18) $$\begin{array}{c} \text{CrossCorr}\left(u,v\right)=\sum_{x}\sum_{y}R\left(x,y\right)I\left(x-u,y-v\right), \end{array}$$

where *R* is the reference image intensity, *I* is the input image intensity, and *x* and *y* represent the partials of images *R* and *I* in *x* and *y*-directions, respectively. The summation is taken over the region (*u*, *v*), where *R* and *I* overlap. When *I*(*x*, *y*) best matches *R*(*x*, *y*), CrossCorr(*u*, *v*) shows the maximum value. 

*Normalized Mutual Information (NMI).* The algorithms of mutual information (MI) have been the most investigated measure for registration of medical image to date. The mutual information of images *I* and *J* is defined by the following [32, 33]:

(19) $$\begin{array}{c} \text{NMI}\left(I,J\;|\;T\right)=\sum_{i,j}P_{i,j}\log\frac{P_{i,j}}{p_{i}p_{j}}, \end{array}$$

where *P*_*i*,*j*_ is the joint probability distribution image of *I* and *J* and *p*_*i*_ and *p*_*j*_ are the marginal probability distribution function of *I* and *J*, respectively. The minimum and maximum values for normalized mutual information are 0 and 1, respectively. 

When images are correctly registered and aligned, there is maximal dependence between the gray values of the images, meaning that the amount of mutual information would be high. Misregistration will cause a decrease in the MI measure [34]. NMI has been used with success for a wide variety of combinations, including MR, CT, SPET, PET, and also time series images [35]. NMI can be found in a large number of studies [34, 36]. 

#### 1.2.3. Optimized Automatic Image Registration 3D

OAIR is a robust image registration algorithm based on FLIRT (FLIRT stands for FMRIB's Linear Registration tool 1.3) [26, 37–39]. The OAIR technique specifies a transformation that minimizes a cost function, which represents the quality of alignment between two images. The method assesses the cost function at the number of different image resolutions, starting with the lowest resolution. Each step of increasing resolution uses the previously specified optimal transformation as the starting point and further refines its values. OAIR method usually works very well with the image of the same modality (e.g., MRI-MRI, CT-CT, and PET-PET). During the OAIR registration, the resampling process will influence the computed value of the cost function; therefore, choosing the best interpolation is important. 

*Outline of the OAIR Method.* (1) The registration algorithm specifies the minimum resolution for each dimension of the target and reference images (they are subsampled by factors two, four, and eight). (2) The reference and target images are interpolated in order to create high resolution isotropic *voxels*. (3) The centers of mass (COM) for the reference and target images are then calculated and one translation level is implemented to align the COM. 

The method uses the right-hand convention in 3D coordinate systems (*X*, *Y*, *Z*) in order to compute the COM. The image origin is generally at the corner of the image (the upper left-hand corner of the image). The axis directions are as follows the *x*-axis goes left to right, the *y*-axis goes top to bottom, and the *z*-axis goes into the image. To compute the COM, the characteristics function of an object in an image is defined by the following

(20) $$\begin{array}{c} b\left(x,y,z\right)=\{\begin{array}{cc} 1, & \text{for}\;\;\text{points}\;\;\text{inside}\;\;\text{of}\;\;\text{the}\;\;\text{image},\\ 0, & \text{for}\;\;\text{points}\;\;\text{outside}\;\;\text{of}\;\;\text{image}. \end{array} \end{array}$$

Next, the area of the image is computed as

(21) $$\begin{array}{c} S=∭b\left(x,y,z\right)dx\;dy\;dz. \end{array}$$

**Figure 5:**
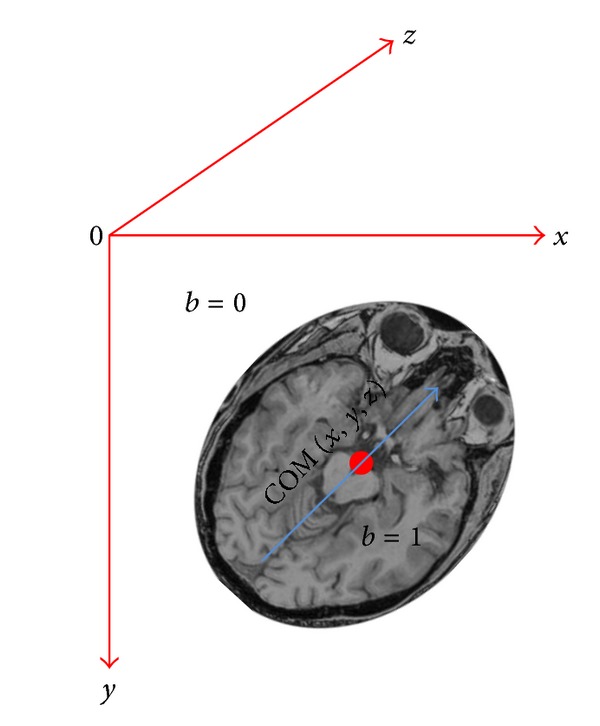
Calculating the COM in the image space; the image origin is in the upper left-hand corner of the image. The *x*-axis goes left to right, the *y*-axis goes top to bottom, and the *z*-axis goes into image.

Figure 5 shows the COM.

The COM, indicated by (*x*_com_, *y*_com_, *z*_com_), is given by the first moments of the object:

(22) $$\begin{array}{c} x_{\text{COM}}=\frac{∭xb\left(x,y,z\right)dx\;dy\;dz}{∭b\left(x,y,z\right)dx\;dy\;dz},\\ y_{\text{COM}}=\frac{∭yb\left(x,y,z\right)dx\;dy\;dz}{∭b\left(x,y,z\right)dx\;dy\;dz},\\ z_{\text{COM}}=\frac{∭zb\left(x,y,z\right)dx\;dy\;dz}{∭b\left(x,y,z\right)dx\;dy\;dz}. \end{array}$$

(4) For each resampled image (which is 8, 4, 2, and 1 times) specifies the transform that minimizes the cost function.

*Optimization Steps. *The theoretical registration problem is completely determined by an interpolation method, a cost function, and a transformation space. However, in practice, an optimization method is needed to find the transformation that minimizes the cost function [26]. In general, all cost functions require global optimization. As a part of the transformation optimization process, the images are subsampled by several factors (e.g., eight, four, and two times) [38]. 

*Levels Eight, Four, Two, and One Optimization*. Reference and target images are interpolated and subsampled by eight, so each image is eight times smaller. The parameters corresponding to the minimum cost function are specified and used as the initial transformation. For the next level (level four) in the optimization, the reference and target images are interpolated and subsampled by four and, like in level eight, the transformation parameters corresponding to the minimum cost function are specified and used as the initial transformation for the next level (level two) in the optimization. For level two optimization, the process repeats, except that the reference and target images are first interpolated and subsampled by factor two. As mentioned above, the parameters of the transformation are systematically varied, and the cost function is assessed for each setting. For level one optimization, 1 mm interpolated images are used and the transformation is generalized to contain 12-DOF. 

The merit of this multiresolution technique is that the initial optimization, at large *n*, has a noticeably reduced computational load, since the number of sample points is considerably less. Additionally, a large subsampling (*n* = 8) uses the lowest resolution image and coarse rotation angle, in which the large features of the image are dominate, and so the overall alignment is easier to find. The following sections explain in detail how each resampled image (which is 8, 4, 2, and 1 times) specifies the transform that minimizes the cost function.

(1)  *Level Eight Optimization.* One of the most difficult tasks in image registration is finding the right orientation or rotation, and most of the erroneous registrations that have been examined have happened primarily because of an incorrect orientation. Thus, the search focuses on the rotational part of the transformation. 

In level eight, the reference image is directed, interpolated, and the cost function is evaluated for coarse rotations angles (−30, −30, −30), (−30, −30, −15),…, (−30, −30, 30), where the values represent the amount of rotation in degrees about *a*, *y*, and *z* axes, respectively. In this case, there would be 125 possible angle configurations, since there are three rotation angles and each angle can contain five different values. A 4-DOF local optimization is also applied to find optimal translation and global scale for each angle configuration. The best 20% of the cost values and corresponding angle configurations (candidate local minima) are stored in vector of minima that is used as starting point for a further optimization, which uses a smaller step size over a narrow range of angles. For each parameter setting related to the top 20% of the cost function minima, the algorithm performs the minima over rotation as well as global scale and translation (previously, the algorithm had not optimized over rotation). For each of these sets of parameters, a 7-DOF optimization is then performed, storing the results of the transformation and cost before and after optimization in a vector of minima. A vector of parameters and top 20% of the min cost function values are considered for the next higher resolution (level four optimization) stage, because the relative costs of each candidate solution may change at higher resolutions. The algorithm also uses interpolation to transform images to this new orientation [26, 37–39].

(2)  *Level Four Optimization. *The algorithm now calls level four with the interpolated images subsampled by 4 to specify the transformation that minimizes the cost function starting with the transformations determined in level eight. The optimization specifies a 7-DOF transformation that corresponds to minimum value of the cost function. This transformation is then perturbed and the cost function is calculated for these new settings. The perturbations correspond to six degrees for each rotation parameter and a global scaling factor of 0.8, 0.9, 1.0, 1.1, and 1.2. A vector of parameters and the top 20% of the cost function minima values are considered for the next step, which involves images subsampled by 2. 

(3)  *Level Two Optimization. *The algorithm uses the images interpolated and subsampled by 2 and computes the value of the cost function for each parameter setting obtained from the level four optimization. It finds the best minimum, and then optimizes it with the 7-DOF, then 9-DOF, and 12-DOF. The algorithm then returns the best minimum after optimization. 

(4)  *Level One Optimization.* The algorithm now level one to use the unsubsampled interpolated images and computes the value of the cost function for each parameter setting obtained from the level two optimization. In this step, one optimization run is performed, with the maximum allowable DOF, as determined by the user (max 12-DOF). The best answer is returned from level one and gives us the minimum cost of differences between the images.

## 2. Materials and Methods

A 3D spoiled gradient recalled (SPGR). MRI of the brain was acquired at Nationwide Children's Hospital of Columbus, Ohio, USA, using a 3T GE MR scanner from a 34-year-old participant. Interpolation techniques were performed on brain scans. Relevant imaging parameters are listed in Table 1. The first initial reference image, also the HR, dimensions were 512 × 512 × 120 (this is native scanner output) with *voxel* size of 0.5 × 0.5 × 1.3 mm^3^ and with slice thickness and spacing between slices of 1.3 mm (acquiring a fully isotropic 3D scan was not feasible because of time, motion artifact, and SNR factors). Because of interpolation and registration time, we simulated the new 3D HR images (simulated reference 2) with a resolution of 256 × 256 × 120 and with a *voxel* size of 1 × 1 × 1.3  mm^3^ and with slice thickness and spacing between slices of 1.3 mm. In the absence of gold standards, simulations are sometimes utilized to assess registration accuracy. A common tactic is to take real data and deform it using appropriate spatial transformation model (affine, rigid, and projective) and other factors that are thought to be relevant in limiting registration accuracy such as simulating the addition of noise and blurring. 

The first low resolution (LR) images were generated from the reference one, and the resolution was decreased (512 × 512 × 60 and with a *voxel *size of 0.5 × 0.5 × 2.6 mm^3^) along the slice direction by subsampling by a factor of 2. The second, third, and fourth LR images were generated from simulated reference 2, and they were subsampled by a factor of 2 in the *x*-, *y*-, and *z* directions. The second LR images were generated with a resolution of 256 × 256 × 60 (axial plane) and with a *voxel* size of 1 × 1 × 2.6 mm^3^. The third LR images were generated with resolution 256 × 128 × 120 (sagittal plane) and with *voxel *size of 1 × 2 × 1.3 mm^3^, and finally, the fourth LR images were generated with a resolution of 128 × 256 × 120 (coronal plane) and with a *voxel* size of 2 × 1 × 1.3  mm^3^. We rotated the LR images in *x*-direction by 5 degrees. Then, we translated the rotated image above in *x* by 2 mm and in *y* by 3 mm. The LR images are corrupted by Gaussian noise (10 standard deviation) and Gaussian blurring (horizontal 5 radius).

Afterward, we used these LR images as input to our interpolation algorithms (trilinear, cubic Lagrangian, quintic Lagrangian, heptic Lagrangian, windowed Sinc, B-spline 3rd order, and B-spline 4th order) to remap to a common size. They were upsampled and back to their original dimension (256 × 256 × 120), and then we compared them to the reference images in order to find the minimum interpolation error during upsampling. Image restoration (adaptive noise reduction and blind deconvolution techniques) was implemented upon the upsampled images to reduce blurring and noise. Adaptive noise reduction algorithm reduces noise without blurring the edges by replacing a pixel value with a weighted sum of all local pixels reached by following a path with small pixel intensity values between neighboring pixels, and blind deconvolution is a method, which allows recovering of the target object from a set of blurred images in the presence or a poorly specified or unknown point spread function (PSF) [40]. Restoration can be implemented by applying any deconvolution method that considers the presence of noise and blurring. 

OAIR was applied on high resolution data set (simulated reference 2) with a resolution of 256 × 256 × 120 and with a *voxel* size of 1 × 1 × 1.3 mm^3^, and the transformed image with a resolution of 256 × 256 × 120 and with a *voxel* size of 1 × 1 × 1.3 mm^3^. Throughout the OAIR, when an optimal fit was achieved, the target image was reformatted using the transformation function and interpolations described above to match the reference image. For achieving a good registration (intensity-based cost function) between the fixed image (reference image) and the moving image (target image), the resampling was essential because the moving image did not necessarily have the same origin, spacing, and number of pixels as the fixed image. Therefore, the resampling process helped us to have the moving image in the grid of the fixed image.

The intensity-based registration method looked for the transformation that would give the smallest value of the cost function, which we assumed was the transformation that also gave the best alignment. During this registration for analyzing the effect of interpolation and cost function, we applied and tested various interpolations and cost functions. The cost functions which were performed in this method included

1. normalized mutual information (NMI);
2. normalized cross correlation (NCC);
3. least squares (LS);
4. correlation ratio (CR).

### 2.1. Image Assessment

There are various ways to evaluate the accuracy of registration technique. They can be divided into qualitative and quantitative methods. For qualitative and quantitative assessment of registered images, we proposed five ways to compare and evaluate the new transformation with the old; we needed to quantify the difference between the geometrically transformed source images with the target image.

#### 2.1.1. Quantitative Assessment

 For the quantitative assessment, we considered a mean squared error (MSE), peak signal to noise ratio (PSNR), and entropy. The MSE and PSNR measures are estimates of the quality of registration images, and entropy is also a suitable choice for quantitative assessment of the accuracy of registration method. 

(1)  *Mean Square Error*. MSE was computed between the original image (reference) and reconstructed image in order to measure the average of the squared difference in image intensities:

(23) $$\begin{array}{c} {\text{SE}}_{ijk}={\left(R_{ijk}-I_{ijk}\right)}^{2}, \end{array}$$

where *i*, *j*, and *k* represent the direct comparison of each coordinate location, *R* is the reference image, and *I* is the reconstructed image. The MSE was computed for 3D brain image in order to assign a value and compare the results.

(24) $$\begin{array}{c} \text{MSE}=\frac{\sum_{i=1}^{n}\sum_{j=1}^{m}\sum_{k=1}^{l}{\text{SE}}_{ijk}}{n\cdot m\cdot l}, \end{array}$$

where *n*, *m*, and *l* are the number of points in the *x*-, *y*-, *z*-directions, respectively, for the reconstructed volume. 

(2)  *Peak Signal to Noise. *PSNR in decibels (dB) between the original image and the registered image is defined by [41]

(25) $$\begin{array}{c} \text{PSNR}=20\times \log_{10}\left(\frac{\text{MAX}}{\text{RMSE}}\right), \end{array}$$

where MAX is the maximum pixel value of the image and RMSE is the square root of the MSE.

(3)  *Entropy.* The desire for a measure of information (commonly termed *entropy*) of a message stems from communication theory [42]. Shannon introduced an adapted measure in 1984 [43], which weights the information per outcome by the probability of that outcome occurring. Given the events occurring with the probabilities, the Shannon entropy is defined as

(26) $$\begin{array}{c} H=\sum_{i}p_{i}\log\frac{1}{p_{i}}=-\sum_{i}p_{i}\log p_{i}, \end{array}$$

where *p* = (histogram  count  in  bin)/total  count. The Shannon entropy can be applied and computed for an image and be used on the distribution of the gray values of the image. An image with a low entropy value has almost a single intensity; it contains very little information. An image with a high entropy value has more or less equal quantities of numerous different intensities; it contains a lot of information [42]. For instance, blurring an image reduces noise and high frequency and thus sharpens the images histogram, resulting in reduced entropy.

#### 2.1.2. Qualitative Assessment

 One way for qualitative assessment is the subtraction of the reference and registered images. Image subtraction techniques can be used to detect subtle changes that may reflect clinically important disease progression [21]. Another way to conduct a qualitative assessment is to create a joint histogram. The joint histogram is a functional tool for visualizing the relationship between the intensities of corresponding *voxels* in two or more images. Visual assessment is also considered for qualitative assessment.

**Figure 6:**
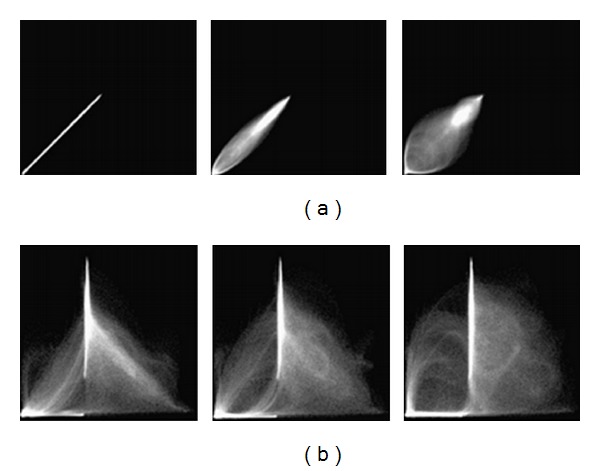
Example  2D histograms for (a) the same MR images of the head, (b) MR and CT images of the head. The left columns show two images when aligned, the middle columns show two image when translated by 2 mm, and the right columns show two images when translated by 5 mm. As can be seen, the joint histogram disperses with increasing mis-registration (obtained from Hill et al. (1994)).

(1)  *Joint Histogram. *The joint histogram is two-dimensional for two grayscale images A and B and is created by plotting the intensity of each *voxel* in image A against the intensity of the corresponding *voxel* in image B. When two images of different modalities are produced, the spatial resolution is likely to be different (see Figure 6). Therefore, before calculating a joint histogram, it is essential to rescale the range of data of the first image to the range of data of the second image. When two images are perfectly aligned, the corresponding anatomical areas overlap, and their joint histogram is highly focused. In misaligned images, anatomical areas are not matched, and they are mixed up and their joint histogram is scattered. For example, the cerebrum region of one image overlaid onto the skull region of another image causes a more dispersed joint histogram. Note, joint histograms are commonly used for different modalities like MR-CT and PET-MR at different stages are investigated in some papers [7, 44]. We implemented the joint histogram technique on the registered images of the same modality for checking the effect of interpolations and cost functions on the accuracy of OAIR.

## 3. Results and Discussion 

**Figure 7:**
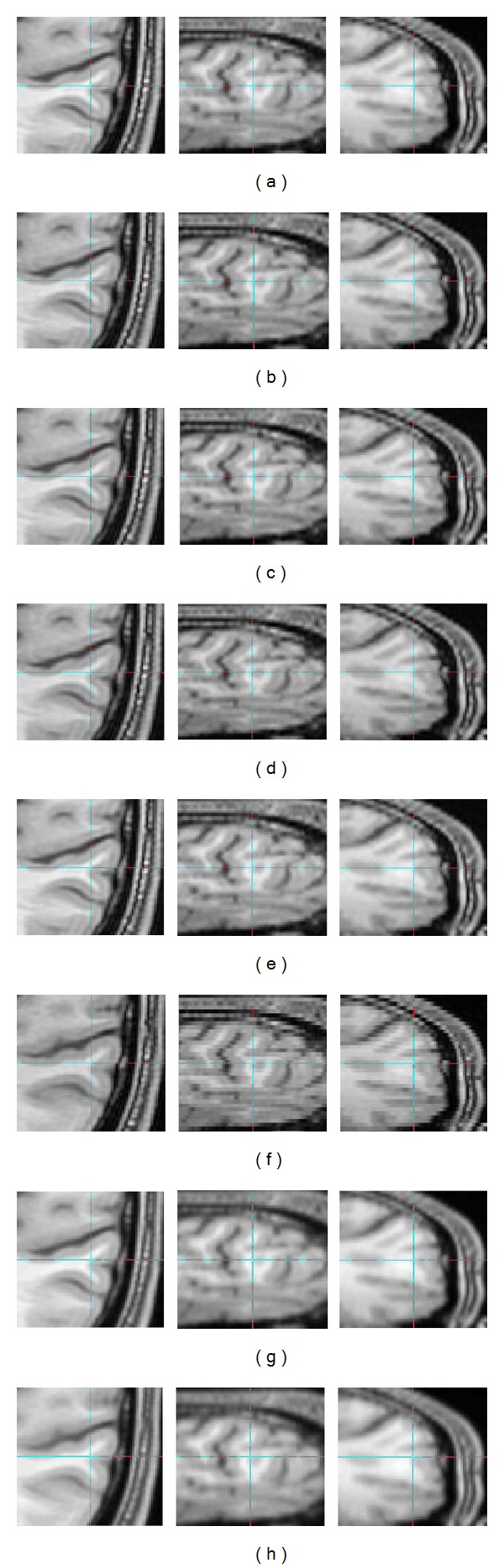
(a) Trilinear, (b) heptic Lagrange, (c) quintic Lagrange, (d) windowed Sinc, (e) cubic Lagrangian, (f) nearest neighbor, (g) B-spline 3rd order, (h) B-spline 4th order, axial view (left column), sagittal view (middle column), and coronal view (right column). These upsampled images are from images that were downsampled by a factor of two in *z*-direction.

In Figure 7, the interpolated images in axial, coronal, and sagittal views are shown for the first LR images. Part (a) illustrates the 3D image interpolated by the trilinear method; in part (b), the image generated by heptic Lagrangian and quintic Lagrangian interpolation images appears in part (c). Part (d), part (e), and part (f) show that 3D images are generated by windowed Sinc, cubic Lagrangian, and nearest neighbor interpolations, respectively. The B-spline 3rd and B-spline 4th interpolations are shown in parts (g) and (h), respectively. The 3D MSEs for 512-sized MRI images were computed in all three planes. To further test the interpolations in 3D, three typical matrix sizes were simulated, namely, 64, 128, and 256. The 3D MSEs of these matrix sizes were tabulated in Table 2. The MSE is inversely proportional to the 3D MRI images size. As a result, the trilinear method yielded more accurate (lower MSE) values than the discussed interpolations.

Also, the interpolated images were quantitatively evaluated by computing the PSNR, which is widely used in the evaluation of reconstructed images [11]. The 3D PSNRs for 64–512 sized MRI images were computed. The 3D PSNRs of these matrix sizes were tabulated in Table 3. The PSNR results for trilinear interpolation for matrix size of 64–512 were approximately 91 (dB), 100 (dB), 111 (dB), and 121 (dB), respectively. Based on Table 3, trilinear interpolation shows PSNR superiority against the other interpolation. In addition, the PSNR was found to slowly increase as the matrix size increased. The second LR images, the matrix size of 256 × 256 × 60 (axial view), the third LR images, the matrix size of 256 × 128 × 120 (sagittal view), and the fourth LR images, the matrix size of 128 × 256 × 120 (coronal view) were simply interpolated separately, and MSE was computed. The results are tabulated in Table 4.

As a result, in Table 4, the interpolated matrix size of 256 × 256 × 60 (axial view) yields more accurate (lower MSE) results than both matrix sizes of 256 × 128 × 120 (sagittal view), 128 × 256 × 120 (coronal view). In other words, the interpolated images in *z*-direction have more quality (lower MSE) than the interpolated images in *x*- and *y*-directions. However, the perceived quality of 128 × 256 × 120 (coronal view) was nearly as good as that of 256 × 128 × 120 (sagittal view).

### 3.1. Visual Quality of Interpolation Techniques

**Figure 8:**
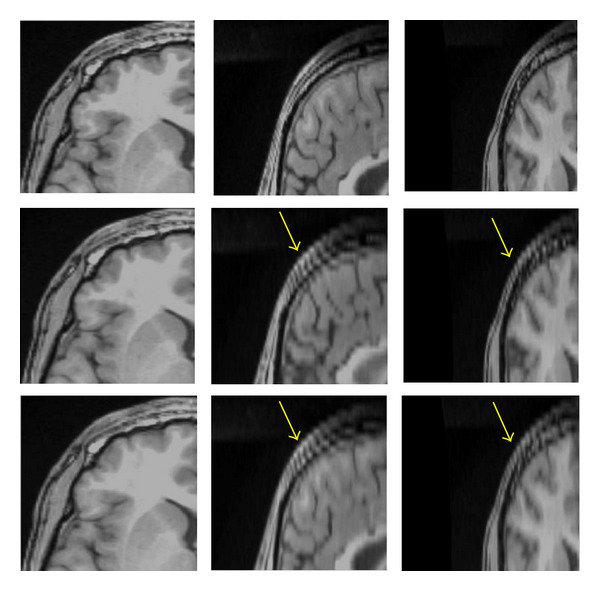
Reference with resolution 256 × 256 × 120 (top row), downsampled by a factor of 2 in the *Z* direction with resolution 256 × 256 × 60 (middle row), interpolated by trilinear (bottom row), axial view (left column), sagittal (middle column), and coronal view (right column). The yellow arrows in sagittal and coronal views show significant jagged-edge distortion.

**Figure 9:**
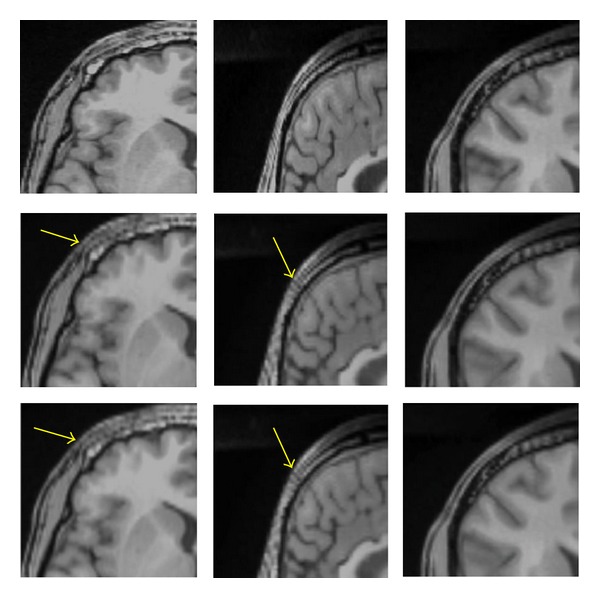
Reference with resolution 256 × 256 × 120 (top row), downsampled by a factor of 2 in the *Y* direction with resolution 256 × 128 × 120 (middle row), interpolated by trilinear (bottom row), axial view (left column), sagittal (middle column), and coronal views (right column). The yellow arrows in axial and sagittal views show significant jagged-edge distortion.

**Figure 10:**
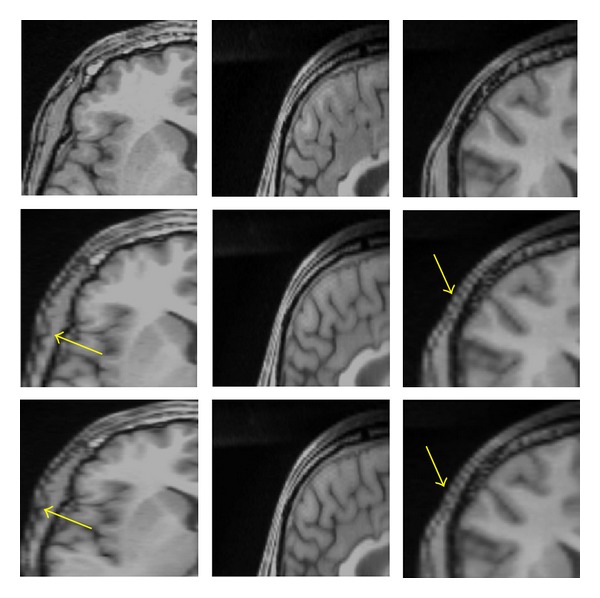
Reference with resolution 256 × 256 × 120 (top row), downsampled by a factor of 2 in the *X* direction with resolution 128 × 256 × 120 (middle row), interpolated by trilinear (bottom row), axial view (left column), sagittal (middle column), and coronal view (right column). The yellow arrows in axial and coronal views show significant jagged-edge distortion.

In Figure 8, we applied the trilinear algorithm on the second LR images, and the resolution in the axial view did not improve because the resolution in *x*- and *y*-directions was already high (256 × 256 × 60) with *voxel* size of 1 × 1 × 2.6 mm^3^, and the resolution in axial direction was constant. In contrast, the resolutions in sagittal and coronal views showed different results, and we saw enough improvement in both planes (interpolated images). As was mentioned before, the resolution in the slice-select direction is lower than plane direction, and we would like to improve the resolution in the slice-selection direction. In Figure 9, we applied a trilinear algorithm on the third LR images with a resolution of 256 × 128 × 120 and a *voxel* size of 1 × 2 × 1.3 mm^3^; the resolution in coronal view did not improve because the resolution in *x*-direction was high. However, the resolutions in sagittal and axial views were changed, and we saw enough improvement in their resolutions. The fourth LR images had a resolution of 128 × 256 × 120 and a *voxel* size of 2 × 1 × 1.3 mm^3^; those results are shown in Figure 10. The resolution in the sagittal view was constant because the resolution in *y* was high and just the sagittal resolution in axial and coronal views was changed. The downsampled results in Figure 8 (middle row) in the sagittal and the coronal views show significant jagged-edge distortion. The trilinear interpolation results in Figure 8 (bottom row) and the coronal views have smoother edges but somewhat blurred appearance overall. Also, the downsampled results in Figure 9 (middle row) in the axial and sagittal views are almost equivalent to the downsampled results in the sagittal and coronal views in Figure 9, and they showed noticeable jagged-edge distortion. The trilinear interpolation results in Figure 9 (bottom row) in the axial and sagittal views had smoother edges but blurred appearance slightly. One can see enough improvement in resolution of both planes (axial and sagittal), but the resolution of the coronal view was constant. The downsampled results in Figure 10 (middle row) were similar to the downsampled results in Figures 8 and 9, but with different perspective (axial and coronal). The trilinear interpolation results in Figure 10 (bottom row) in axial and coronal views showed enough improvement in their planes, but the sagittal view was constant. 

### 3.2. Runtime Measurement

The runtimes of the various interpolation schemes were computed on MR images with a resolution of 256 × 256 × 60 (axial view). In the axial view, the nearest neighbor was the fastest interpolation, with a runtime of 16.8 s, and the trilinear was a bit slower than the nearest neighbor with a runtime of 20.4 s. Cubic Lagrangian was fairly fast (35.7 s) and required less time than the quintic Lagrangian, heptic Lagrangian, and windowed Sinc, with 75.2 s, 159.6 s and 182.3 s, respectively.

**Figure 11:**
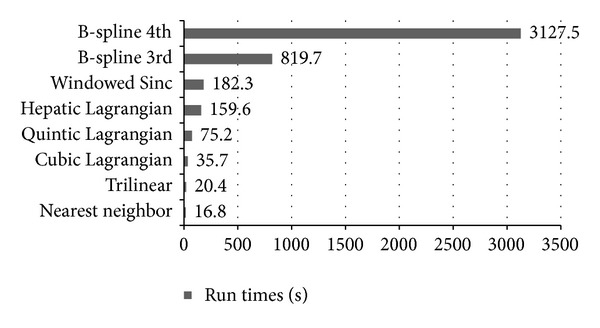
Run times measured on the Intel Xeon with 2.13 GHz 2 processor. Among the discussed interpolation techniques, the trilinear is one of the fastest interpolations and runs quickly, with 20.4 s.

Interpolation with the B-spline 3rd order and B-spline 4th order took about 48 and 185 times as long as nearest neighbor interpolation. This weak performance was caused by evaluation of the exponential function necessary to specify the weights and increasing the order of B-spline showed the interpolation drastically. The results of the run times are presented in Figure 11.

Among the interpolations techniques discussed, the trilinear method was one of the fastest techniques and had the smallest interpolation error. The nearest neighbor had a strong point in which the original *voxel* intensities were preserved, but the resulting image was degraded significantly and had a blocky appearance. Our experiments showed that the heptic Lagrangian technique had smaller error than the quintic Lagrangian and the cubic Lagragian. The windowed Sinc had smaller error than the nearest neighbor, B-spline 3rd order, and B-spline 4th order. The main drawback of windowed Sinc interpolation was that, it generated significant ripple artifacts in the surrounding of image edges. The B-spline 3rd order and B-spline 4th order were the slowest techniques in this study, and B-spline 3rd order produced one of the worst results in terms of similarity to the original image. These results demonstrated that the increment of the order in B-spline will not significantly improve the interpolation quality, and this will just magnify the edge effects and the degree of blurriness, which already noticeable when compared to trilinear and Lagrangian methods. The theory and application of B-spline were analyzed by researchers [45, 46], and they found the third-order B-spline interpolator to be sufficient for some specific practical applications [47]. Currently, we believe that the trilinear can offer the best compromise between speed and accuracy in upsampling. 

### 3.3. Analyzing the Effect of Interpolation Techniques on Accuracy of Cost Functions-Based OAIR Algorithm

**Figure 12:**
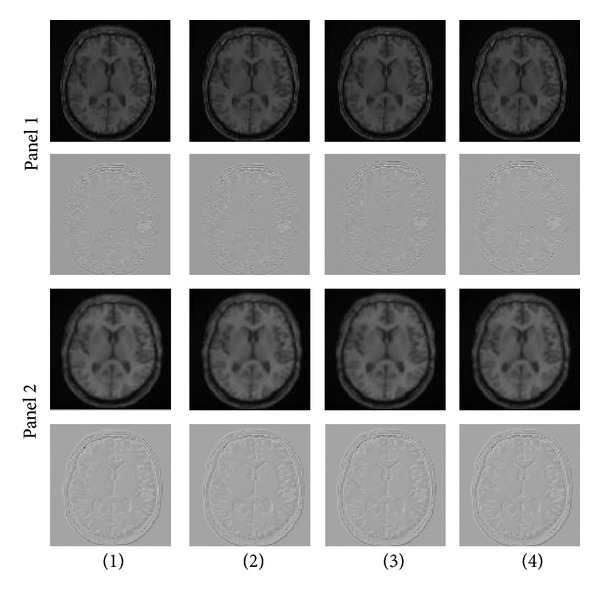
In the top panel one, axial slices from two unregistered 3D MRI volumes using an intensity-based cost function (CR (first column), LS (second column), NCC (third column), and NMI (fourth column)) are shown. Windowed Sinc interpolation was used during registration for the panel one. The second row of the panel one shows subtraction of the images (registered and reference images). The panel two shows similar axial slices from the same two data sets after registration of the full 3D volumes using the same intensity-based cost function (CR (first column), LS (second column), NCC (third column), and NMI (fourth column)). B-spline 3rd-order interpolation was used during registration for the panel two. The second row of the panel two shows subtraction of the images. Although differences of the first rows of panel one and panel two are not easily observed by eye, subtraction of the images shows that differences are present, as seen on the second rows of panel one and panel two.

**Figure 13:**
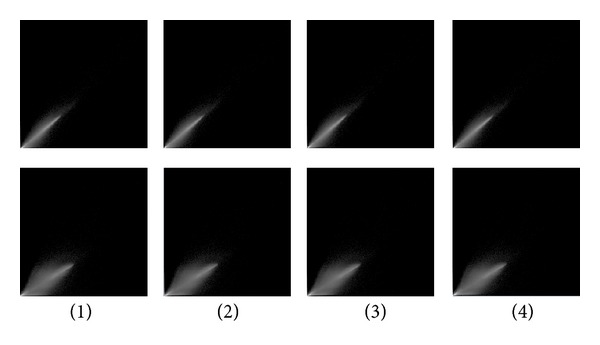
Joint histogram for registered 3D MRI volumes using an intensity-based cost function (CR (first column), LS (second column), NCC (third column), and NMI (fourth column)) are shown. The top row is generated from the images when registered using windowed Sinc interpolation, and the bottom row is generated from the images when registered using B-spline 3rd-order interpolation.

 We implemented OAIR 3D described in Section 1 to perform registration between images, and seven interpolation techniques using similarity measures NCC, LS, CR, and NMI. We computed MSE and PSNR of our results, and the experimental results are listed in Table 5. It is important to note that the interpolation error during upsampling (before registration) is different than the interpolation error of geometric transformation (during registration). For instance, the interpolation algorithm, which has remarkable performance in upsampling process, may have insufficient performance in geometric transformation [48]. Statistical analysis of Table 5 showed that there was insignificant difference between the sets of image registered using CR, LS, NCC, and NMI (*P* value > 0.9994 for all cost functions). However, the effect of interpolation was considerable, and we observed significant difference between the sets of image registered using different interpolations (*P*-value < 0.0001 for all interpolations). For instance, sets of images registered using windowed Sinc interpolation were significantly better than sets of images registered using B-spline 3rd-order interpolation with similar cost functions (lower MSE and higher PSNR). For qualitative assessment, we investigated the accuracy of registered results using intensity-based cost functions (CR, LS, NCC, NMI). Windowed Sinc and B-spline 3rd-order interpolations were used during registration (other interpolations schemes can also be used if more investigation is desired).  Figure 12 shows axial slices from two registered 3D MRI volumes with their subtractions. The panels show axial slices from two data sets (3D simulated images with a resolution of 256 × 256 × 120 and with a *voxel* size of 1 × 1 × 1.3 mm^3^) after registration of the three-dimensional volumes using an intensity-based CR (first column), LS (second column), NCC (third column), and NMI (fourth column). Windowed Sinc (panel one) and B-spline 3rd-order (panel two) interpolations were used as resampling. In the second row of the panels, after registration, the pixel intensities of the reference and target images were roughly identical and different images were considerably smooth. In both panels, although differences of registered 3D MRI volumes using an intensity-based CR (first column), LS (second column), NCC (third column), and NMI (fourth column) were difficult to see by visual inspection, changing the interpolation showed that significant differences between two panels exist. In the panel two, where B-spline 3rd order was used during registration, the boundary of the skull, which was masked out of the images during registration, could still be observed easily in the difference images, whereas in the panel one, where windowed Sinc was used during registration, the skull in different images was not easily detected by eye. A possible reason for checking the effect of interpolations and cost functions for registration can be seen by visual inspection of the joint histogram in Figure 13, which contains several histograms of 3D MRI using an intensity-based CR (first column), LS (second column), NCC (third column), and NMI (fourth column). In the top and bottom rows, windowed Sinc and B-spline 3rd-order interpolations were used during registration, respectively. The top row in Figure 13 showed the joint histogram for 3D images at registration using windowed interpolation and with small amount of misregistration, and there was a diagonal in the distribution with the small dispersion. However, in the bottom row, the B-spline 3rd-order interpolation led to large mis-registration of the image, and increased off-diagonal entries started to appear, and the distribution became more dispersed. The distribution of the bottom row is nonsymmetric, and so the linear relationship is not preserved. In general, the intense inhomogeneity will noticeably change for different interpolations; this is one of the reasons that will induce nonsymmetric dispersion and a nonlinear relationship between intensities. However, the change in the appearance of the histograms for these 3D MRI volumes using CR, LS, NCC, and NMI is *insignificant*. We used these joint histograms to better understand the effect of different interpolations and cost functions during registration. We also measured joint entropy of the registered images using various interpolations and cost functions. Because joint entropy is directly related, the joint probability distribution described the statistical relationship of corresponding *voxel* intensities. Entropy increased with increasing mis-registration as can be seen in visual appearance of the joint histogram (see Figure 13). High dispersion of the joint histogram is equivalent to high joint entropy [49]. The joint entropy results are shown in Table 6, and there are no significant differences between the entropies of registered images using CR, LS, NCC, and NMI (*P*-value ≥ 0.9999 for all cost functions), which means that they have the similar dispersion in the joint histogram, but there were significant differences between the entropies of registered images using different interpolations (*P*-value < 0.0001 for all interpolations). Also, we computed costs for different *voxel* similarity cost functions that were used in registration with different interpolations and cost functions. We used inverse cost functions; thus, the minimum cost function corresponded to better registration. The results are shown in Table 7, and the registered images using CR, LS, NCCI, and NMI yielded very close results (*P*-value ≥ 0.9999 for all cost functions), whereas the registered images using different interpolations yielded different results (*P*-value ≤ 0.0041). 

Statistical analysis of Tables 5, 6, and 7 showed that interpolations had a* significant *effect on the registration accuracy, whereas cost functions had no effect on the registration accuracy.

## 4. Conclusion

Interpolation techniques play a critical role in the improvement and deterioration of the quality of the image as the resolution changes. Thus, the interpolation error is crucial in assessing the interpolation techniques. The interpolation error depends on features such as geometric deformation and the content of the image; therefore, only one evaluation method would not be adequate to evaluate all properties of an algorithm, and a variety of methods should be applied. The comparison is performed by visual quality assessment, quantitative interpolation error determination, and run time measurement. In this study, the results of the algorithms showed that the trilinear method had the smallest interpolation error and the highest PSNR and was one of the fastest techniques, making it appropriate for upsampling in 3D MR images and super resolution. Although super computers are able to compute a huge amount of data in real time, fast methods might be required for online resampling of image sequences or films [50]. The resulting images for trilinear interpolation were less smooth and blocky than other interpolated images. Nevertheless, trilinear interpolation has the effect of losing some high frequency information from the image [51]. 

Also, the effect of cost functions (LS, NMI, NCC, and CR), and interpolations (trilinear, cubic Lagrange, quintic Lagrange, heptic Lagrange, windowed Sinc, B-spline 3rd order, and B-spline 4th order) for OAIR of 3D brain images was examined, and our experimental results showed that interpolations can effectively decrease or increase the failure possibility of the registration algorithm, and the robustness of method was not due to the choice of cost function, but the choice of interpolation was critical on the robustness of registration. In addition, each component of the optimization method was also necessary to achieve the accurate registrations. Studying the precise effect of transformation on registration is an active research area [52] but is beyond the scope of this paper.

We noted that the study presented in the tables and figures relied on the same modality (MRI); in addition, these results are not representative of different modality combinations. Perhaps other conclusions would be obtained by the use of different modality combinations or transformations, for instance, MRI-PET or CT-MRI. 

Also, OAIR was explained in detail, and it was a powerful image registration algorithm. This algorithm was a fully automated algorithm and proposed various resampling interpolation methods combined with CR, LS, NCC, and NMI as a cost function. One of the advantages of this method was using a feature detector (corners are used as the features) to automatically choose a large number of potentially matchable feature points in both images. The algorithm is able to detect identical features in all projections of the scene regardless of the particular image deformation. This method has direct potential for registering clinical MRI images. We have validated this method quantitatively and qualitatively, on the simulated and real data, respectively. Also, there are many excellent sources for more in-depth discussions of image registration that the reader may wish to read and learn [53–56].

## 5. Future Work

We would like to study the precise effect of transformation on registration, and we are also interested in combining the information of three MRI plane orientations using brain images in order to increase the resolution of 3D brain image based on super resolution reconstruction (SRR) technique.

## Figures and Tables

**Table 1:** Imaging parameters associated with 3D reference and low-resolution images.

3D images	No. of slices	Matrix size	*Voxel* size (mm^3^)
Reference 1	120	512 × 512	0.5 × 0.5 × 1.3
Simulated reference 2	120	256 × 256	1 × 1 × 1.3
Low resolution 1	60	512 × 512	0.5 × 0.5 × 2.6
Low resolution 2	60	256 × 256	1 × 1 × 2.6
Low resolution 3	120	256 × 128	1 × 2 × 1.3
Low resolution 4	120	128 × 256	2 × 2 × 1.3

**Table 2:** 3D MSE for MR images of 64–512 for interpolations of trilinear, nearest neighbor, B-spline 3rd order, B-spline 4th order, cubic Lagrangian, quintic Lagrangian, heptic Lagrangian, and windowed Sinc.

	MSE			
	Matrix size			
Interpolation	64 × 64	128 × 128	256 × 256	512 × 512
Trilinear	0.22683	0.03088	0.00282	0.00024
Nearest neighbor	0.75852	0.09615	0.01295	0.00135
B-spline 3rd order	0.50714	0.06435	0.00706	0.00095
B-spline 4th order	0.32088	0.05742	0.00618	0.00075
Cubic Lagrangian	0.24283	0.04956	0.00554	0.00027
Quintic Lagrangian	0.24204	0.03286	0.00552	0.00026
Heptic Lagrangian	0.24181	0.03284	0.00549	0.00025
Windowed Sinc	0.24338	0.05123	0.00559	0.00027

**Table 3:** 3D PSNR for MR images of 64–512 for interpolations of trilinear, nearest neighbor, B-spline 3rd order, B-spline 4th order, cubic Lagrangian, quintic Lagrangian, heptic Lagrangian, and windowed Sinc.

	PSNR (dB)			
	Matrix size			
Interpolation	64 × 64	128 × 128	256 × 256	512 × 512
Trilinear	91.95	100.61	111.01	121.76
Nearest neighbor	86.78	95.76	104.47	114.29
B-spline 3rd order	87.62	96.57	106.17	114.73
B-spline 4th order	89.44	96.92	106.60	115.93
Cubic Lagrangian	91.73	98.63	108.14	121.29
Quintic Lagrangian	91.74	100.42	108.16	121.45
Heptic Lagrangian	91.75	100.44	108.19	121.61
Windowed Sinc	91.72	98.49	108.11	121.28

**Table 4:** 3D MSE for MR image of matrix sizes of 256 × 256 × 60, 256 × 128 × 120, and 128 × 256 × 120 for interpolations of trilinear, nearest neighbor, B-spline 3rd order, B-spline 4th order, cubic Lagrangian, quintic Lagrangian, heptic Lagrangian, and windowed Sinc (compared to 3D simulated reference 2 with resolution 256 × 256 × 120).

	MSE		
	Matrix size		
Interpolation	256 × 256	256 × 128	128 × 256
Trilinear	0.002793	0.002820	0.002983
Nearest neighbor	0.014754	0.016352	0.016544
B-spline 3rd order	0.010866	0.013058	0.012870
B-spline 4th order	0.006383	0.007063	0.007319
Cubic Lagrange	0.004519	0.005546	0.005213
Quintic Lagrange	0.004504	0.005530	0.005195
Heptic Lagrange	0.004132	0.005524	0.005192
Windowed Sinc	0.004605	0.005660	0.005285

**Table 5:** The 3D MSE and PSNR for registered image of 256 × 256 × 120 using interpolations of trilinear, B-spline 3rd order, B-spline 4th order, cubic Lagrange, quintic Lagrange, heptic Lagrangian, windowed Sinc, and cost functions of CR, LS, NCC, and NMI. The affine transformation contains 12 DOF and was implemented during registration.

Interpolation	Cost function	MSE	PSNR (dB)
Trilinear	Correlation ratio	0.023505	115.32
Trilinear	Least squares	0.023591	115.16
Trilinear	Normalized cross-correlation	0.023597	115.10
Trilinear	Normalized mutual information	0.023623	115.09
B-spline 3rd order	Correlation ratio	0.065592	106.30
B-spline 3rd order	Least squares	0.065732	106.27
B-spline 3rd order	Normalized cross-correlation	0.064311	106.34
B-spline 3rd order	Normalized mutual information	0.065588	106.31
B-spline 4th order	Correlation ratio	0.037540	111.37
B-spline 4th order	Least squares	0.037597	111.36
B-spline 4th order	Normalized cross-correlation	0.037631	111.35
B-spline 4th order	Normalized mutual information	0.037604	111.36
Cubic Lagrange	Correlation ratio	0.020723	116.35
Cubic Lagrange	Least squares	0.020887	116.28
Cubic Lagrange	Normalized cross-correlation	0.020891	116.27
Cubic Lagrange	Normalized mutual information	0.020793	116.32
Quintic Lagrange	Correlation ratio	0.019982	116.76
Quintic Lagrange	Least squares	0.020108	116.71
Quintic Lagrange	Normalized cross-correlation	0.020082	116.72
Quintic Lagrange	Normalized mutual information	0.019979	116.76
Heptic Lagrange	Correlation ratio	0.019588	116.86
Heptic Lagrange	Least squares	0.019770	116.78
Heptic Lagrange	Normalized cross-correlation	0.019736	116.79
Heptic Lagrange	Normalized mutual information	0.019639	116.83
Windowed Sinc	Correlation ratio	0.019119	117.06
Windowed Sinc	Least squares	0.019190	117.02
Windowed Sinc	Normalized cross-correlation	0.019329	116.96
Windowed Sinc	Normalized mutual information	0.019160	117.04

**Table 6:** The joint entropy for the registered images of matrix size of 256 × 256 × 120 using interpolations of trilinear, B-spline 3rd order, B-spline 4th order, cubic Lagrange, quintic Lagrange, heptic Lagrange, windowed Sinc, and cost functions of CR, LS, NCC, and NMI. Their entropies were achieved using mutual information.

Interpolation	Cost function	Entropy
Trilinear	Correlation ratio	2.3723
Trilinear	Least squares	2.3718
Trilinear	Normalized cross-correlation	2.3704
Trilinear	Normalized mutual information	2.3787
B-spline 3rd order	Correlation ratio	2.4499
B-spline 3rd order	Least squares	2.4486
B-spline 3rd order	Normalized cross-correlation	2.4489
B-spline 3rd order	Normalized mutual information	2.4529
B-spline 4th order	Correlation ratio	2.3874
B-spline 4th order	Least squares	2.3889
B-spline 4th order	Normalized cross-correlation	2.3891
B-spline 4th order	Normalized mutual information	2.3881
Cubic Lagrange	Correlation ratio	2.3649
Cubic Lagrange	Least squares	2.3613
Cubic Lagrange	Normalized cross-correlation	2.3615
Cubic Lagrange	Normalized mutual information	2.3636
Quintic Lagrange	Correlation ratio	2.3527
Quintic Lagrange	Least squares	2.3559
Quintic Lagrange	Normalized cross-correlation	2.3534
Quintic Lagrange	Normalized mutual information	2.3544
Heptic Lagrange	Correlation ratio	2.3549
Heptic Lagrange	Least squares	2.3561
Heptic Lagrange	Normalized cross-correlation	2.3511
Heptic Lagrange	Normalized mutual information	2.3517
Windowed Sinc	Correlation ratio	2.3425
Windowed Sinc	Least squares	2.3452
Windowed Sinc	Normalized cross-correlation	2.3434
Windowed Sinc	Normalized mutual information	2.3443

**Table 7:** The CR, MI, NMI, and NCC of reference and registered images. The 3D registered images using trilinear, B-spline 3rd order, B-spline 4th order, cubic Lagrange, quintic Lagrange, heptic Lagrange, windowed Sinc interpolations, and cost functions of CR, LS, NCC, and NMI.

Registration		Pixel similarity cost functions			
Interpolation	Cost function	Correlation ration	Mutual information	Normalized mutual information	Normalized cross-correlation
Trilinear	Correlation Ratio	0.030921	0.572625	0.758732	0.016443
Trilinear	Least square	0.030943	0.575558	0.758843	0.016472
Trilinear	Normalized cross-correlation	0.030978	0.577968	0.758742	0.016498
Trilinear	Normalized mutual information	0.030874	0.571644	0.758756	0.016452
B-spline 3rd	Correlation ratio	0.091192	0.906945	0.789837	0.047106
B-spline 3rd	Least square	0.091371	0.905995	0.790105	0.047204
B-spline 3rd	Normalized cross-correlation	0.089311	0.905822	0.788936	0.046135
B-spline 3rd	Normalized mutual information	0.091554	0.906234	0.789860	0.047101
B-spline 4th	Correlation ratio	0.047523	0.645413	0.764792	0.026291
B-spline 4th	Least square	0.047664	0.647675	0.764965	0.026334
B-spline 4th	Normalized cross-correlation	0.047837	0.646529	0.764938	0.026347
B-spline 4th	Normalized mutual information	0.047741	0.646291	0.764879	0.026328
Cubic Lagrange	Correlation ratio	0.027451	0.557755	0.738121	0.014453
Cubic Lagrange	Least square	0.027678	0.559016	0.738517	0.014556
Cubic Lagrange	Normalized cross-correlation	0.027751	0.557785	0.738253	0.014558
Cubic Lagrange	Normalized mutual information	0.027632	0.558993	0.734889	0.014486
Quintic Lagrange	Correlation ratio	0.026226	0.533578	0.731717	0.013931
Quintic Lagrange	Least square	0.026307	0.538433	0.730597	0.014006
Quintic Lagrange	Normalized cross-correlation	0.026227	0.533604	0.731881	0.013986
Quintic Lagrange	Normalized mutual information	0.026234	0.532218	0.731289	0.013912
Heptic Lagrange	Correlation ratio	0.025983	0.537712	0.729622	0.013651
Heptic Lagrange	Least square	0.026274	0.532524	0.731221	0.013767
Heptic Lagrange	Normalized cross-correlation	0.026273	0.531087	0.730581	0.013741
Heptic Lagrange	Normalized mutual information	0.026162	0.531199	0.730681	0.013672
Windowed Sinc	Correlation ratio	0.024201	0.263132	0.720291	0.013392
Windowed Sinc	Least square	0.025253	0.260997	0.720884	0.013445
Windowed Sinc	Normalized cross-correlation	0.024727	0.266973	0.720306	0.013544
Windowed Sinc	Normalized mutual information	0.024819	0.262372	0.730032	0.013426
